# Age distribution of accidents in hospitals affiliated to Iranian Ministry of Health

**Published:** 2019-08

**Authors:** Mohamad Hajijafari, Fateme Asgarian, Mohamad Hosein Ziloochi

**Affiliations:** ^*a*^Trauma Research Center of Kashan, Kashan University of Medical Sciences, Kashan, Iran.

## Abstract

**Background:**

Accidents, as the third important cause of mortality, are among main challenges of communities due their costs and irreversible damages. This study aimed to investigate the age distribution of people referred to the hospitals affiliated by Iran’s ministry of health because of accidents.

**Methods:**

In a cross-sectional study, using a CDC checklist, data related to the injured people who referred to the hospitals affiliated by Iran’s ministry of health in a one year period was gathered from hospital records, as well as interview and observation. The collected data was analyzed by descriptive and analytical statistics in SPSS11 software.

**Results:**

The commonest age group involved in accidents during the year 1395 (2016) was middle-aged (37%) followed by youth (25%). The commonest accidents in children were: fall (23%), traffic accidents (19%), and burn (13%). In adolescents, the commonest accidents were: traffic accidents (32%), fall (16%), and violence and poisoning (5%). The commonest accidents in youth were: traffic accidents (35%), violence (9%), and poisoning (6%). In middle-aged group, the commonest accidents were: traffic accidents (30%), fall (12%), and violence and poisoning (7%). The commonest accidents in elderly were: fall and traffic accidents (25%), burn (4%), and violence and poisoning (3%).

**Conclusions:**

Making plan and policies to prevent accidents and injuries is a health priority and a key tool for improving the safety of the community. Also, informing the all age groups of the community about preventive actions is necessary.

**Keywords:**

Road Traffic Injury, Trauma, Hospital

